# Realizing a Novel Friction Stir Processing-Enabled FWTPET Process for Strength Enhancement Using Firefly and PSO Methods

**DOI:** 10.3390/ma13030728

**Published:** 2020-02-05

**Authors:** Senthil Kumaran S, Jayakumar Kaliappan, Kathiravan Srinivasan, Yuh-Chung Hu, Sanjeevikumar Padmanaban, Srinivasan N

**Affiliations:** 1 Department of Manufacturing Engineering, School of Mechanical Engineering, Vellore Institute of Technology, Vellore 632 014, Tamil Nadu, India; senthilkumaran.s@vit.ac.in; 2 School of Computer Science and Engineering, Vellore Institute of Technology, Vellore 632 014, Tamil Nadu, India; jayakumar.k@vit.ac.in; 3 School of Information Technology and Engineering, Vellore Institute of Technology (VIT), Vellore 632 014, Tamil Nadu, India; kathiravan.srinivasan@vit.ac.in; 4 Department of Mechanical and Electromechanical Engineering, National ILan University, No. 1, Sec. 1, Shenlung Rd., ILan City 26041, ILan County, Taiwan; 5 Center for Bioenergy and Green Engineering, Department of Energy Technology, Aalborg University, 6700 Esbjerg, Denmark; san@et.aau.dk; 6 School of Mechanical Engineering, Vellore Institute of Technology, Vellore 632 014, Tamil Nadu, India; srinivasan.narayanan@vit.ac.in

**Keywords:** carbon nanotube (CNT), firefly algorithm (FFA), friction welding of tube to tube plate using an external tool (FWTPET), friction stir processing (FSP), particle swarm optimization (PSO)

## Abstract

The friction welding of tube to tube plate using an external tool (FWTPET) is widely deployed in several industrial applications, such as aerospace, automotive, and power plants. Moreover, for achieving a better tensile strength and hardness in the weld zone, the friction stir processing (FSP) technique was incorporated into the FWTPET process for joining aluminum alloys (AA6063 tube, AA6061 tube plate). Furthermore, it has to be noted that FWTPET was applied for joining the AA6063 tube to the AA6061 tube plate, and FSP was deployed for reinforcing the weld zone with carbon nanotube (CNT) and silicon nitride (Si_3_N_4_) particles, thereby attaining the desirable mechanical properties. Subsequently, the Taguchi L_25_ orthogonal array was used for identifying the most influential input and output FWTPET + FSP process parameters. Furthermore, particle swarm optimization (PSO) and the firefly algorithm (FFA) were deployed for determining the optimized input and output FWTPET + FSP process parameters. The input process parameters include CNT, Si_3_N_4,_ rotational tool speed, and depth. Furthermore, the tensile strength of the welded joint was considered as the output process parameter. The process parameters predicted by PSO and FFA were compared with the experimental values. It was witnessed that deviation between the predicted and experimental values was minimal. Moreover, it was found that FFA provided a superior tensile strength prediction than PSO.

## 1. Introduction

Welding is defined as one of the manufacturing processes in which similar/dissimilar materials can be joined with or without the application of pressure. In general, the welding process is classified as (i) fusion welding or (ii) solid-state welding. Fusion welding is a joining technique that uses heat to join base materials or workpieces. The primary issues associated with fusion welding are (i) the formation of deleterious phases, (ii) the generation of residual stresses and distortion, (iii) the generation of high heat input when joining dissimilar welds with a different melting point and coefficient of thermal expansion. Friction welding (FW) [1,2], friction stir processing (FSP) and friction stir welding (FSW) [3], on the other hand, are essential solid-state welding processes with significant applications in the power plant industry. Friction welding is a type of solid-state welding process for joining symmetrical shapes, such as tube to tube or rod to rod, without melting of the workpiece [4,5]. Friction stir welding is a type of solid-state welding process to join the plate to a symmetrical shape without melting of the workpiece by applying force with the help of an external tool. In our work, friction stir processing is deployed for improving the mechanical properties by reinforcing the suitable materials (carbon nanotube (CNT) + Si_3_N_4_) in the weld zone.

Friction welding of tube to tube plate using an external tool (FWPET) + FSP is a method of improving the mechanical properties of welding materials by using an external tool. The tool progresses toward the tube plate during rotation, and the heat generated during solid-state welding is considerably less than that during fusion welding. The tool creates a sufficient amount of force on the tube to tube plate and produces an adequate amount of plastic deformation at the weld interface. FWTPET + FSP is a microstructure refinement process which also improves the properties at the weld zone of the workpieces. It is primarily used for modifying the microstructure of near-surface layers [6]. Friction stir welding (FSW) is also used to join two metallic materials without melting the workpiece which uses a non-consumable tool similar to FWTPET [7,8]. The combined process of FWTPET and FSP was developed to join the unsymmetrical shapes of the tube and tube plate. In addition, FSP is based on the principle of friction stir welding (FSW). The advantage of the combined process of FWTPET and FSP is that it is a simple and environmentally friendly technique capable of altering microstructure by recrystallization. The FWTPET and FSP process is capable of producing leak-proof joints that are more efficient and eco-friendlier than many other conventional welding processes [9,10]. This method has several implications including modification of the microstructure of a material due to severe plastic deformation, which leads to super-plasticity. Thus, the unique feature of FWTPET and FSP has several advantages, such as the possibility of mass production, increased safety of welders, and compliance with health-related regulations [11,12,13,14,15]. Another advantage of using FWTPET is that enhanced mechanical properties by consuming minimum energy are evident, as discussed by the researchers [16,17,18,19,20,21,22].

Nature-motivated algorithms such as the metaheuristic algorithm (MA) are most widely used for optimization as they have many advantages over conventional algorithms. MAs are diverse, including genetic algorithm, simulated annealing, ant and bee algorithm, particle swarm optimization (PSO) [23,24,25,26], harmony search algorithm (HSA) [27], and cuckoo search.

The main impact of recent research work is the further improvement of the tensile strength of the tube to tube plate joint when compared with the FWTPET process. The novel idea of the FWTPET + FSP process focuses on achieving desirable properties around the weld zone through the infiltration of reinforcement in the base metal during the welding of tube to tube plate using an external tool set-up. The main objective is to enhance the design life of components such as box type heat exchangers and radiators. Furthermore, the PSO and FFA are also applied to optimize suitable process parameters that yield better tensile strength [28,29,30,31,32].

The combination of FWTPET + FSP techniques is proposed to overcome the issue of retention or increase in tensile strength. It is reported that specimens joined through FWTPET + FSP achieved better tensile strength along with enhanced mechanical properties. Furthermore, this was also experimentally shown through the tensile test. The hardness at the region around the weld is always higher than that around the base metal. Moreover, the combination of FWTPET + FSP increases the tensile strength in the weld zone.

In this work, the incorporation of FWTPET and FSP is carried out to weld two dissimilar aluminum alloys, AA6061 tube plate to AA6063 tube, using carbon nanotube (CNT) and Si_3_N_4_ particles as reinforcements, added via ex situ approaches by varying the processing parameters (speed of tool, depth of cut, and percentage of reinforcements). Although research groups widely explored the FWTPET and FSP as independent techniques across the world, the combined process of FWTPET + FSP was not explored widely by researchers. Furthermore, CNT and Si_3_N_4_ particles were introduced for reinforcement of the joining location of the Al6061 tube plate and Al6063 tube for achieving the desirable properties.

## 2. Materials and Methods

**Figure 1:**
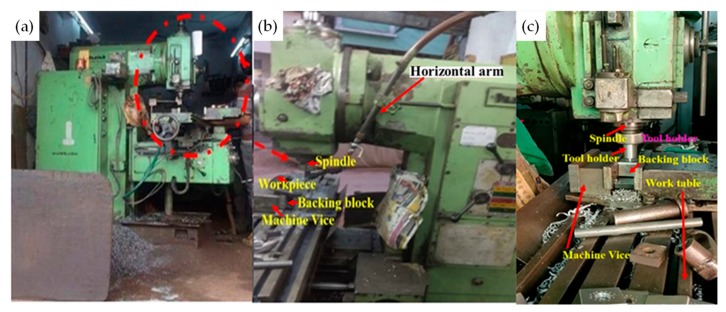
Experimental set-up used for the friction welding of tube to tube plate using an external tool (FWTPET) + friction stir processing (FSP) technique: (**a**) vertical milling machine (VMM); (**b**,**c**) magnified view indicating components of VMM.

**Figure 2:**
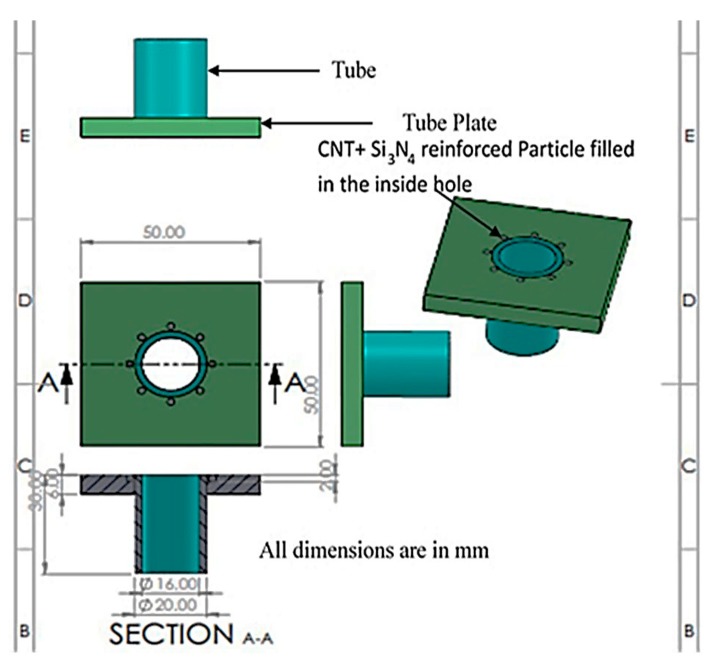
The actual dimensions of the tube and tube plate specimens for implementing the FWTPET + FSP technique.

A vertical milling machine (VMM) (Figure 1), with a slight modification for performing FWTPET + FSP, was used in this study, and the exact specification/ model is provided in Table 1. FWTPET + FSP was developed in-house (vice, fixture, tool arbor, and tool), and it is a welding process suitable to join the tube to the tube plate. The coordinates (X- and Y-movement) of VMM are regularly calibrated for positional errors. The magnified view illustrates the FWTPET process of tube (AA6063) and tube plate (AA6061) specimens in this study. Prior to the welding process, these specimens were cleaned from unwanted contaminants and dirt. The detailed information about the FWTPET process, the tools, and the actual dimension of the specimens is depicted in Figure 2.

**Figure 3:**
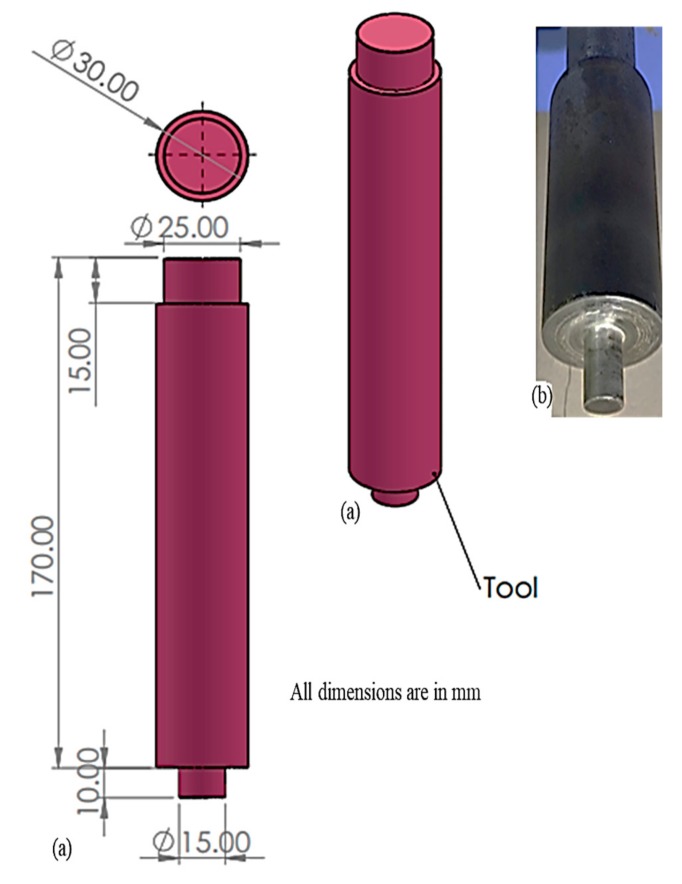
The FWTPET + FSP processing tools: (**a**) dimensions; (**b**) photograph.

**Figure 4:**
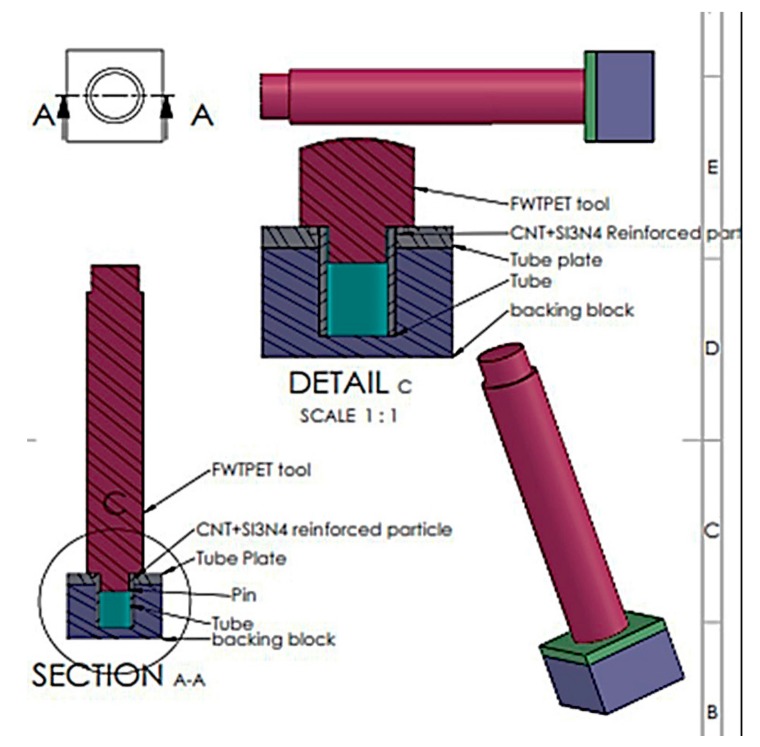
Schematic diagram—FWTPET + FSP arrangement of the tube to tube plate weld set-up.

**Figure 5:**
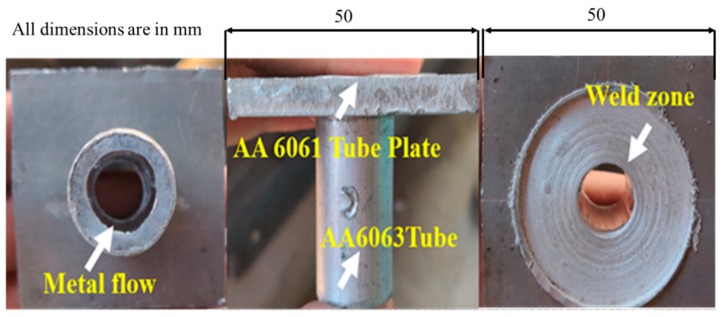
Pictorial view—welded joint of AA6061 (tube plate) and AA6063 (tube) using FWTPET + FSP technique.

Tungsten carbide was used as the FWTPET + FSP processing tool, which is portrayed in Figure 3. The schematic diagram for the FWTPET + FSP arrangement of the tube to tube plate weld set-up is illustrated in Figure 4. Furthermore, this exemplifies the joining of the AA6063 tube and AA6061 tube plate by reinforcing the materials of CNT and Si_3_N_4_ in the weld zone with the help of the tungsten carbide rotational tool. The filling of reinforced particles was done using a predetermined hole of 2 mm on the top surface of the tube plate. Moreover, as the tool continuously rotated based on the input process parameters, it mixed the reinforced CNT and Si_3_N_4_ particles, which were filled in the drilled holes. The continuous rotation of the tool helped in achieving a homogeneous distribution of CNT and Si_3_N_4_ particles in the tube plate weld zone using FWTPET + FSP. Furthermore, the plastic flow of the metals progressed toward the center of the tool axis, and a forging force was simultaneously applied to the weld zone of the tube to tube plate. The tool was withdrawn after a predetermined time. Moreover, Figure 5 shows the welded joint of AA6061 (tube plate) and AA6063 (tube) using the FWTPET + FSP technique. Also, the regions of metal flow and weld zone are indicated. Furthermore, the welded joint was subjected to characterization analyses such as tensile strength, Vickers microhardness, scanning electron microscopy (SEM), and X-ray diffraction (XRD). The input process parameters considered in the FWTPET + FSP investigation were rotational tool speed (rpm), depth of cut (mm), and the weight percentages of carbon nanotube (CNT) and Si_3_N_4_.

The Vickers microhardness tester (LecoTM LM 300, VIT Vellore, Tamil Nadu, India) AT model was used to measure the hardness of the base metal, and a 500-g load was applied to the welded specimen through a diamond pyramid indenter. Furthermore, an average of 10 measurements was taken from different regions. The programs for SEM (Hitachi SEM, VIT Vellore, Tamil Nadu, India) and XRD (XPert^3^, VIT Vellore, Tamil Nadu, India) analysis that were deployed include the Pro suite and Advanced SAXS (XPert^3^, VIT Vellore, Tamil Nadu, India) data analysis. The applied SEM working parameters include acceleration voltage (20 keV), the working distance (26 mm), and the secondary electron image (SEI) (Hitachi SEM, VIT Vellore, Tamil Nadu, India). The incident scanning electron beam applied over the welded specimen spawned various signals; however, only the secondary electrons were filtered. It can be witnessed that each of these signals were sensitive to different aspects of the specimen, and they might provide diverse information about the specimen. The notable point is that the different signals were generated from various regions in the specimen.

X-ray diffraction (XRD) analysis was performed on the welded specimen for identifying the different reinforced particles. The XRD equipment was operated at 40 kV and 30 mA with Cu-anode Ka radiation and a step time of 2 s. A beam of X-rays directed at the welded specimen interacted with the electrons. Furthermore, the electrons oscillated under the influence of the incoming X-rays and became the secondary source of electromagnetic radiation. The secondary radiation traveled in all directions. The waves emitted by the electrons had the same frequency, as the incoming X-rays were coherent. The emission could undergo constructive or destructive interference.

The Universal Testing Machine (UTM) (MCS, VIT Vellore, Tamil Nadu, India) set-up was used to calculate the tensile strength of the tube and tube plate welded specimen. Moreover, the Taguchi method, which employs the concept of an L_25_ orthogonal array (OA), was used in the FWTPET + FSP technique. In total, 25 experiments were conducted, based on the various levels of input process parameters such as CNT, Si_3_N_4_, rotational tool speed, and depth. Also, optimization methods such as PSO and FFA were employed in the FWTPET + FSP technique. In this research work, the result was ensured by successfully repeating the experiment at least three times (three independent welded specimens) applied to the L_25_ OA. Additionally, PSO and FFA were applied for selecting the optimized process parameters in the FWTPET + FSP technique, in order to achieve a better tensile strength of the AA6061 tube plate (RI, VIT Vellore, Tamil Nadu, India) to AA6063 tube welded specimen.

## 3. Results and Discussion

**Figure 6:**
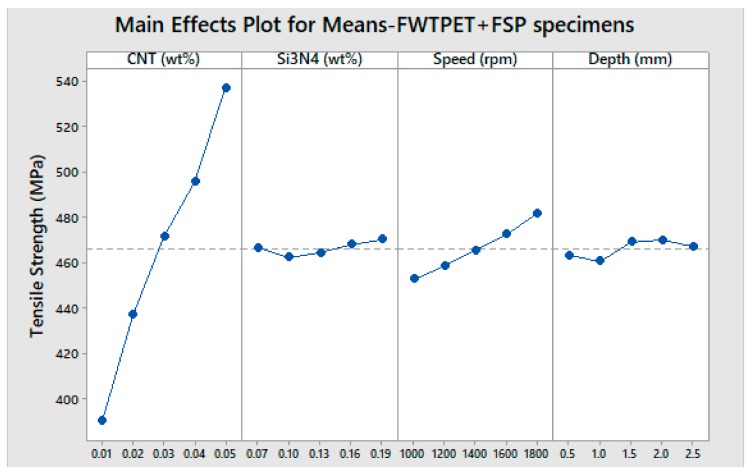
The main effect plot (means) for FWTPET + FSP specimens.

**Figure 7:**
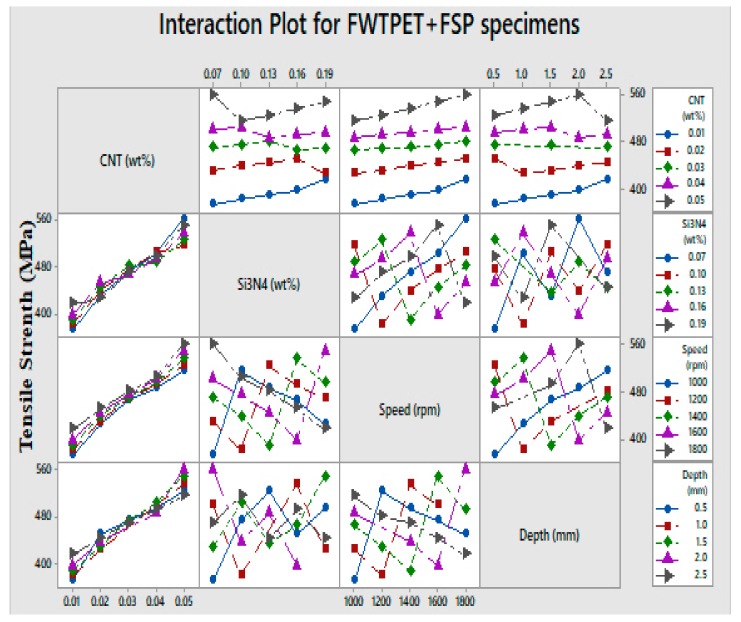
Interaction plots for FWTPET + FSP specimens.

The tensile strength of the welded specimen is a vital factor for critical manufacturing components in several industries [33,34]. Specifically, box type heat exchangers, components (cans) and aluminum collectors in automobile engines and aluminum evaporators, as well as central heating boilers, are some of the applications where the FWTPET + FSP technique might be extensively used in the future. Hence, the selection of FWTPET + FSP process parameters was critical as it determined the quality of the welded joint. In this work, an L_25_ orthogonal array was used for determining the most influential FWTPET + FSP input process parameters. Herein, the input process parameters included CNT, Si_3_N_4_, rotational tool speed, and depth, as indicated in Table 2. The boundary conditions that were used to run the PSO and FFA are listed in Table 3. The boundary conditions refer to the lower and upper limits of each input process parameter such as CNT, Si_3_N_4_, rotational tool speed, and depth. The main effect plot (means) for FWTPET + FSP specimens is illustrated in Figure 6. Furthermore, interaction plots for FWTPET + FSP specimens are depicted in Figure 7. Moreover, it can be witnessed from Table 2 that CNT (0.05 wt.%), Si_3_N_4_ (0.07 wt.%), speed (1800 rpm), and depth (2.0 mm) yielded the highest tensile strength of 560 MPa.

An optimization algorithm (OA) is a process that employs an iteration to find an optimum solution for an engineering problem, particularly in the areas of manufacturing and mechanical design. Furthermore, with the advances in computing technology, optimization is now vital for any generic engineering problem. Soft computing approaches such as fuzzy logic, deep learning, evolutionary computation, optimization algorithms, pattern regeneration, differential algorithms, and metaheuristics provide a better solution. When a proper OA is implemented for an engineering problem, savings in cost and an increase in performance of the product are achieved. Frequently, a compromise of quality is achieved through the trial-and-error method by engineers/technologists. A more scientific way of addressing this issue is to employ an optimization algorithm [35].

In manufacturing, design process optimization refers to enhancing the desired output, simultaneously decreasing the desired variation. Since innovations and new process technologies are becoming more sophisticated in the domain of manufacturing, particularly in the area of the welding, sheet metal forming was developed to address the uncertainty and approximation issues. PSO was developed in 1995 by Eberhart and Kennedy and it is an algorithm that optimizes and iteratively offers a solution. PSO was developed after inspiration from the social behavior of bird flocking. Yang proposed the metaheuristic firefly algorithm in 2008.

Furthermore, researchers in this field optimized the submerged arc welding parameter using the GA and PSO. In another work, the issue of spiking was addressed during electron beam welding (EBM) of an ETP copper plate using PSO and GA. There were other studies by different researchers for solving assembly sequence planning using FFA. Furthermore, the different variants of FFA were designed/discussed by the researchers for different applications including traveling salesman, job shop scheduling, assembly sequence planning and tolerance optimization problems.

In this work, the two different optimization techniques, i.e., PSO and FFA, were applied for achieving the optimized process parameters that resulted in better tensile strength for the FWTPET + FSP technique. The PSO algorithm starts its search and offers accurate solutions by updating generations through continuous iterations. It works on the intelligence technique exhibited by animals or birds in a group, for instance, schools of fish or flocks of birds. The food searching technique used by swarms is incorporated in PSO. The optimization problem is described as determining the best solution from the population of potential solutions.

The swarms generally use their own learning experience and also get input from neighbors while searching for food. Each particle carries a potential solution for the optimization problem and a velocity *V*. The PSO is a population-based stochastic algorithm inspired by the social behaviors of living species, such as bird flocking. To reach the food at position *Z*, each bird moves at a distance on the plane with the velocity *v*, and then the bird decides the next movement distance and speed based on its current best position. This process is related to an optimization problem, where the optimal solution is indicated as food, and each bird in the flock is related to an N particle with possible solution and velocity. The potential solution for the optimization problem is calculated by randomly setting the input parameter value R, and its velocity is also assigned randomly. At each generation, the particle is updated with new values. The best value reached by individual particles is maintained in the best position. The overall best solution reached by the particle population is the best position. The parameter inertia weight *w* is added to improve the search; with a higher value of *w*, a global search is done, and, with a lower value, a local search is carried out. However, *w* has to be high in the beginning and it has to decrease with an increase in generation. To improve the performance further, the chi parameter is added. Here, *r1* and *r2* are random values within the range 0–1, whereas *c1* and *c2* are set to 2.0. Using Equation (1), the new velocity *V*’ value of the particle is calculated.

(1) $$V^{\prime }\left[p\right]=chi\times w\times r1\times c1\times \left(pBestPosition\left[p\right]-R\left[p\right]\right)+r2\times c2\times \left(gBestPosition\left[i\right]-R\left[p\right]\right))$$

The new parameter values *R’ [p]* are found using Equation (2).

*R′ [p] = R[p] +V’ [p]*. (2)

**Figure 8:**
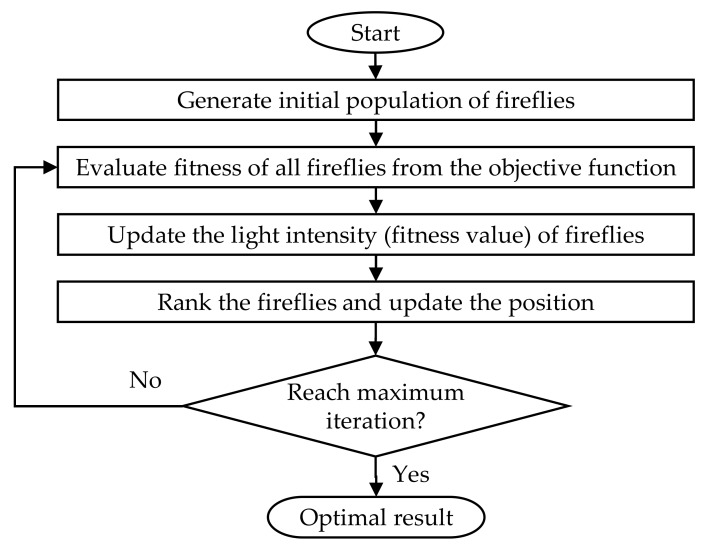
Flowchart of firefly algorithm.

The flash behavior of fireflies inspired the firefly algorithm. The flashing lights of the firefly or the bioluminescence are usually generated by the chemicals present in the lower abdomen of the firefly. The steps involved in the firefly algorithm are illustrated in Figure 8. It can be observed that the firefly uses its flashes or luminance to attract prey and mates, as well as to defend itself from enemies. The rules deployed in the firefly algorithm design are as follows:

(1) All fireflies are unisex; thus, the attractiveness is based only on the brightness.
(2) The fireflies with lower brightness move toward the higher-brightness fireflies.
(3) The fireflies with higher brightness move randomly.

The objective function output represents the brightness of the fireflies. For example, in a maximization optimization problem, the fireflies with lower object function values are attracted to the fireflies with higher objective function values. The best firefly, which has the maximum objective function value, starts moving randomly [5]. The firefly algorithm initially creates *n* fireflies. Each firefly *X* contains two components; one includes the design parameter values and the other is the output obtained when these parameter values are applied to an objective function. The number of design parameters is represented as d. The parameter values are stored in *P*. The objective function is termed as *f*. Initially, the brightness of *n* fireflies is calculated. For each firefly, the design parameters are assigned with random values and passed to the objective function, and its output is stored in *X*. Each firefly in the *n* firefly group is compared. When the brightness of the *i*-th firefly is higher than the *j*-th firefly, the *j*-th firefly design parameters are adjusted to reach the *i*-th firefly. The distance between the fireflies is calculated using Equation (3),

(3) $$D=\overset{d}{\underset{k=0}{\sum }}{\left(P_{i}\left[k\right]-P_{j}\left[k\right]\right)}^{2}$$

Then, the attractiveness value is calculated using Equation (4).

(4) $$Attractiveness=P_{O}\times e^{\left(-gamma*D*D\right)}$$

where *P_O_ is the brightness* at *r = 0, and gamma is the light absorption coefficient*. The *j*-th firefly’s new parameter values are found using Equation (5).

*P_i_ [k] = P_i_[k] + attractiveness(P_j_[k]-P_i_[k]) + alpha × (rand-0.5)*. (5)

The best firefly is found, and its parameter values are adjusted randomly using Equation (6).

*P_best_[k] = P_best_[k] + alpha × rand*. (6)

The first step in the optimization problem is to identify the design parameters. The design parameters chosen should be minimum and should play a significant role in the optimization process. In the second step, the optimization problem has to be represented as a mathematical model. The input parameters chosen were CNT, Si_3_N_4_, depth, and speed. The framed objective function is given in Equation (7).

*Tensile strength* = 300.79 + 3514 *(CNT)* + 44.0 *(Si3N4)* + 0.03583 *(speed)* + 2.68 *(depth)*. (7)

The output value of the objective function represents the light intensity or attractiveness of the fireflies in the firefly algorithm. In the case of the PSO algorithm, the output of the objective function is its potential solution. When the parameter values are adjusted to reach the brighter fireflies or best particles, there is a possibility that new parameter values can be above or below the limit. In order to bring the parameter values within the limit, the normalization function given in Equation (8) is used.

*P_i_[k] = (Limitmax[k] − Limitmin[k]) × (P_i_[k] − baseMin[k])/(baseMax[k] − baseMin[k]) + Limitmin[k]*. (8)

Two experiments were conducted; one was done through the PSO algorithm and another one was done using the firefly algorithm, and the results of these two algorithms are discussed in the next two subsections.

### 3.1. Result Using PSO Algorithm

The PSO algorithm initially decides the number of particles, number of generations, and the minimum and maximum velocity. The particle count (*N*) was set to 100, and the generation count (*NG*) was set to 1000. The minimum and maximum velocities were stored as *VMax* and *VMin*. The maximum and minimum values of the four input parameters were stored in arrays *RMax* and *RMin*, respectively. The input parameter value array *R* was populated with randomly generated values within the range *RMin* and *RMax* of that parameter. With this *R*-value, the output tensile strength was calculated using Equation (7), whereby the *R*-value and its output formed a particle. Thus, 100 particles were constructed in this way. In every generation, for every particle *P*, the particle velocity *V*` was updated. Based on the velocity, new parameter values *R*` were calculated, and the output tensile strength *M[P]* was determined with *f(R`)*. If *M[P]* was higher than the particle’s previous best (*PBest*) or global best (*GBest*), then *PBest* and *GBest* were updated. this was repeated for 1000 generations, and *GBest* gave the maximum output tensile strength obtained from this optimization algorithm. The pseudocode for this result is given in Appendix A. The values initialized for PSO parameters, such as agent particles, iteration count, inertia weight, and learning weight for this optimization problem, are detailed in Table 4.

In Experiment I, the optimization function designed using an L_25_ orthogonal array was applied in the PSO algorithm. Initially, to create 100 particles, the randomly generated input parameter values were passed to the optimization function, and the output tensile strength value was calculated. Afterward, the velocity adjustment was made in each particle in every generation based on the global best and its previous best. The update of the global best was done in every generation. After 1000 generations, the global best value for output tensile strength was found to be 547.10 MPa.

### 3.2. Result Using Firefly Algorithm

The number of fireflies (*n*) was initialized as 20. The total number of generations (*N*) was set to 100. The minimum and maximum values of the four design parameters, CNT, Si_3_N_4_, depth, and speed, were stored in a lower and upper array, respectively. The gamma value was set to 1.0 and the alpha value was set to 0.02. The array *PR* was populated with random values generated within the range of lower and upper of that parameter. The initial set of 20 fireflies was created by applying the randomly generated values of design parameters for the objective function *f* given in Equation (7), whereas this function returned the output tensile strength, *v*. Subsequently, these 20 firefly values were compared. When the output tensile strength of the *i-th* firefly was higher than that of the *j-th* firefly, the difference between the parameter values was used to determine the attractiveness value. The new attractiveness value was calculated using Equation (5). Then, it was checked whether the new parameter values were within the range; if not, the values were normalized using Equation (8). Subsequently, *PR* was applied in Equation (7), which determined the output tensile strength.

If this new output tensile strength was more significant than the best firefly (BestFR), then BestFR was updated with this value. The *i*-th firefly input parameter was randomly initialized, and output tensile strength was calculated. If this output was more significant than BestFR, then it was updated. The process was repeated for 100 iterations. The BestFR value at the end of the 100 iterations was the maximum output tensile strength achieved using the firefly algorithm. The pseudocode for this result is given in Appendix B. The firefly algorithm parameters like firefly count, generation count, and gamma and alpha values used in the experiment are detailed in Table 5.

Experiment II was conducted to find the best value for the input parameter in order to get the maximum output tensile strength. For experiment II, the optimization function served as a fitness function for the firefly algorithm. An initial set of 20 fireflies was created by populating the input parameter with random values within the parameter range. Then, these 20 fireflies were compared for 100 generations. The input parameter values of fireflies with lower output were adjusted according to fireflies with higher outputs, and this was carried out for 100 generations. After 100 generations, the best firefly gave an output tensile strength of 904.37 MPa.

### 3.3. Comparison of the Results Obtained Using PSO and Firefly Algorithms with the Experimental Values

**Figure 9:**
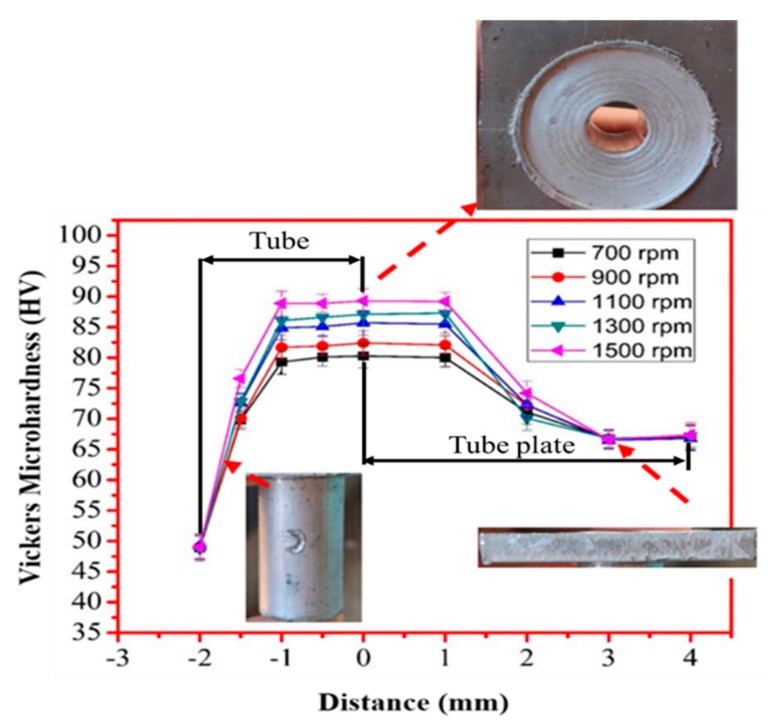
The typical Vickers microhardness profile of specimen subjected to the FWTPET + FSP technique.

By comparing the results of Experiments I and II, it was found that the firefly algorithm performed better than PSO for this objective function. The best values for the input parameters CNT, Si_3_N_4_, speed, and depth found in Experiment I (PSO) and Experiment II (firefly) are detailed in Table 6. FFA gave a peak tensile strength value of 904.7 MPa. However, with the PSO algorithm, a peak tensile strength value of 547.10 MPa was obtained. Table 6 provides a comparison between the experimental and predicted values of the tensile strength. Figure 9 is based on the tensile test performed on the experimental values of the tube to tube plate weld joint (see Table 2).

Furthermore, Table 6 represents the optimized parameters for finding the maximum tensile strength. Using the predicted optimum values of PSO and FFA, the validation experiment was conducted using the achieved input parameters. It can be witnessed that there was a minimal deviation between the predicted and experimentally gained values for tensile strength, which confirmed the hands-on viability of the PSO and firefly algorithm for the FWTPET + FSP process. Hence, a maximum possible tensile strength was attained using the firefly optimization. The tensile strength achieved using the optimized process parameters was 910 MPa (0.05 CNT, 0.19 Si_3_N_4,_ 1700 rpm tool rotation speed, and 1.4 mm depth). The firefly algorithm gave better results than PSO. A monotonic increment of Vickers microhardness (Figure 9) was noted for the rotational tool speed for the base of the metal region to the weld zone.

**Figure 10:**
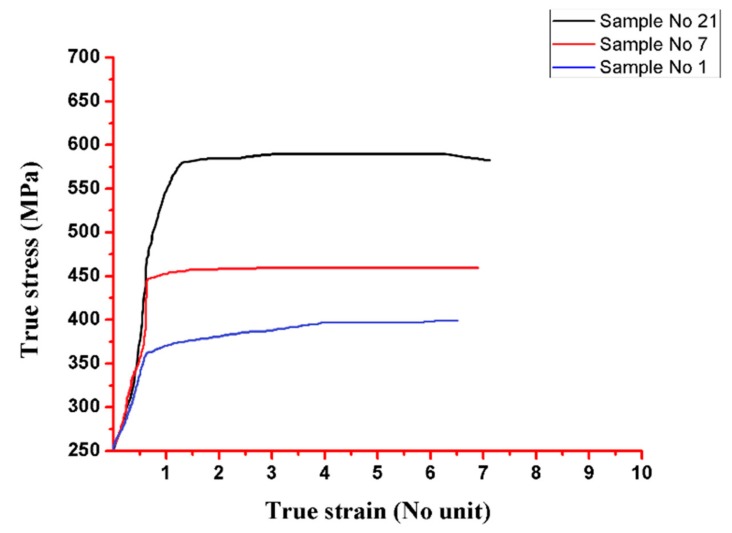
True stress and true strain of three different (sample No. 1 = 372 MPa tensile strength, sample No. 7 = 438 MPa tensile strength, and sample No. 21 = 560 MPa tensile strength) specimens subjected to FWTPET + FSP technique.

**Figure 11:**
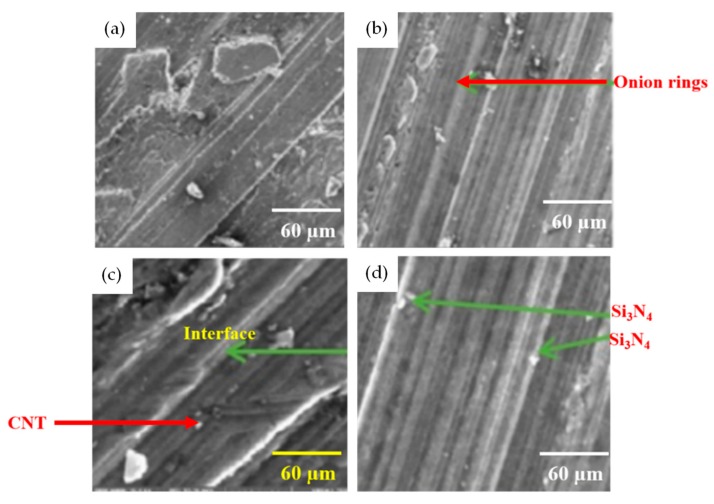
SEM images of specimens after FWTPET + FSP technique: (**a**) sample 1 (1000 rpm tool rotational speed); (**b**) sample 7 (1400 rpm tool rotational speed); (**c**) sample 16 (1600 rpm tool rotational speed); (**d**) sample 21 (1800 rpm tool rotational speed).

**Figure 12:**
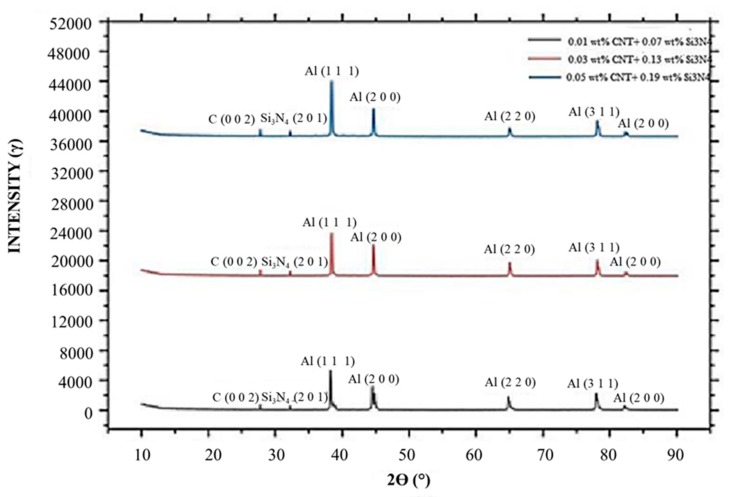
The 2θ X-ray diffraction (XRD) scan of FWTPET + FSP specimens with different reinforcement volumes of CNT and Si_3_N_4_.

Furthermore, the hardness was not compromised after FWTPET + FSP in the weld zone. At this juncture, it is to be noted that base materials AA6061 and AA6063 exhibited lower Vickers microhardness. In general, an increase in Vickers microhardness was noted in all regions compared to base materials with a load of 500 g (Figure 9). The tensile strength was predicted as per the design of the experimental Taguchi L_25_ orthogonal array given in Table 2. It was found experimentally that a higher tensile strength resulted from a rotation speed and a depth of cut of 1800 rpm and 2.0, respectively, at 0.05 (wt.%) CNT and 0.07 (wt.%) Si_3_N_4_. Furthermore, in our work, samples No. 21, 7, and 1 were selected for their tensile strength (Figure 10). These specimens (samples No. 21, 7, and 1) exhibited onion rings (Figure 11b), showing a higher strength. Furthermore, a fine disbursement of reinforcements was revealed in Figure 11a–d by scanning electron microscopy (SEM). These reinforcements were detected by XRD measurement (Figure 12). The specimens joined by FWTPET + FSP (refer to specimen 21 in Table 2) exhibited an ultimate tensile strength of 560 MPa. The weld metal strength was higher than that of the base metal (Al alloy).

The XRD patterns of the Al base metal and CNT- and Si_3_N_4_-reinforced particle samples are presented in Figure 12. The peaks of the aluminum alloy and CNT and Si_3_N_4_ reinforcement were detected in the composite substrate material around the weld zone. The initial dislocation density of Al/CNT and Si_3_N_4_ after FWTPET + FSP was evaluated based on both the microstrain and the crystallite size using the Williamson–Hall plot obtained from the XRD patterns obtained at room temperature (XRD; CN2301; Rigaku, Japan) with a Cu-Kα radiation source (λ = 1.5405 Å). The scanning range, rate, and step size for the XRD analysis were 20–100°, 1°∙min^−1^, and 0.02°, respectively. The XRD pattern of the Al/CNT- and Si_3_N_4_-reinforced particle sample indicated the presence of the substrate material, although some of the peaks were depressed. The Al crystallized into the CNT and Si_3_N_4_ cubic structure with typical diffraction peaks. The two main diffraction peaks of CNT and Si_3_N_4_ corresponded to the (002) and (201) planes. The expected peak of (002) and (201) planes was observed with different weight percentages of 0.01, 0.03, and 0.05 CNT and 0.07, 0.13, and 0.19 Si_3_N_4_, respectively. Furthermore, the increases in wt.% of the CNT- and Si_3_N_4-_reinforced particle in the weld zone proved that there was an increase in the hardness and tensile strength of the FWTPET + FSP weld specimen.

## 4. Conclusions

- In the present study, the FWTPET + FSP technique was used to join a tube to tube plate, representing a novel and state-of-the-art process, which is extensively deployed in several industrial applications. Therefore, to achieve a reasonable manufacturing cost, investigators are discovering novel welding processes in order to attain substantial development in the weld joint properties. This process is capable of welding the Al 6063 tube to Al 6061 tube plate with weld joints of excellent quality, featuring enhanced mechanical and metallurgical properties.
- The aluminum alloy AA6061 tube plate and AA6063 tube were joined successfully using the FWTPET + FSP technique with different reinforcements (CNT and Si_3_N_4_), while different weight percentages of CNT and Si_3_N_4_ were added to the weld zone. The measured quantity of the reinforcements was placed in the pre-drilled hole on the AA6061 tube plate.
- The optimization of the process parameters was carried out using the Taguchi method. The optimized values of CNT, Si_3_N_4_, rotational tool speed, depth, and ultimate tensile strength were 0.05 wt.%, 0.07 wt.%, 1800 rpm, 2 mm and 560 MPa, respectively.
- We optimized the process parameters of the FWTPET + FSP technique by applying the PSO and FFA algorithms for predicting the optimum process parameters.
- The algorithmic results indicated that the firefly algorithm provided better tensile strength prediction than the PSO technique. The firefly algorithm predicted the tensile strength as 904.37 MPa, while PSO predicted the tensile strength as 547.10 MPa. Furthermore, this result was confirmed by experiments (tensile strength), and the deviation between experimental and algorithmic results was found to be minimal. The FWTPET + FSP specimen (processed at a rotational tool speed of 1700 rpm, with 0.05 wt.% CNT, 0.19 wt.% Si_3_N_4_, and a depth of 1.4 mm) exhibited a tensile strength of 910 MPa.

## Figures and Tables

**Table 1:** Specification of the vertical milling machine used for the FWTPET + FSP technique.

Machine Type	Vertical Milling Machine
Make (manufacturer)	ALTO V40W VMC
Table size (mm × mm)	1350 × 310
Spindle motor power	5.5 kW
Spindle speed (m/min)	45–2500 rpm
Feed (mm/min)	16–800

**Table 2:** Design of experiments as per the Taguchi L_25_ orthogonal array. CNT—carbon nanotube.

Experiment	CNT (wt.%)	Si_3_N_4_ (wt.%)	Speed (rpm)	Depth (mm)	Tensile Strength (MPa)
1	0.01	0.07	1000	0.5	372
2	0.01	0.10	1200	1.0	380
3	0.01	0.13	1400	1.5	388
4	0.01	0.16	1600	2.0	396
5	0.01	0.19	1800	2.5	415
6	0.02	0.07	1200	1.5	429
7	0.02	0.10	1400	2.0	438
8	0.02	0.13	1600	2.5	443
9	0.02	0.16	1800	0.5	450
10	0.02	0.19	1000	1.0	425
11	0.03	0.07	1400	2.5	470
12	0.03	0.10	1600	0.5	474
13	0.03	0.13	1800	1.5	480
14	0.03	0.16	1000	1.5	465
15	0.03	0.19	1200	2.5	468
16	0.04	0.07	1600	1.0	501
17	0.04	0.10	1800	1.5	504
18	0.04	0.13	1000	2.0	486
19	0.04	0.16	1200	2.5	492
20	0.04	0.19	1400	0.5	495
21	0.05	0.07	1800	2.0	560
22	0.05	0.10	1000	2.5	515
23	0.05	0.13	1200	0.5	524
24	0.05	0.16	1400	1.0	536
25	0.05	0.19	1600	1.5	548

**Table 3:** Boundary conditions used for implementing particle swarm optimization (PSO) and firefly algorithm (FFA) approach.

Parameters Bounding Condition	Parameters
0.01 ≤ A ≤ 0.05	CNT
0.07 ≤ B ≤ 0.19	Si_3_N_4_
1000 ≤ C ≤ 1800	Speed
0.5 ≤ D ≤2.5	Depth

**Table 4:** Initial parameters of the PSO algorithm.

Input Parameters	Value of Parameters
Number of agent particles	500
Number of iterations	500
Maximum permissible inertia weight Wmax	0.9
Minimum permissible inertia weight Wmin	1.2
Maximum defined learning rate C1max = C2max	2
Minimum defined learning rate C1min = C2min	2

**Table 5:** Initial parameters of the firefly algorithm.

INPUT Parameters	Value of Parameters
Number of fireflies	20
Number of generations	100
Gamma	1.0
Alpha	0.02

**Table 6:** Comparison between the values predicted using various algorithms (PSO and FFA) with the experimental values of the FWTPET + FSP technique.

Predicted/Experimental Values	CNT (wt.%)	Si_3_N_4_ (wt.%)	Speed (rpm)	Depth (mm)	Tensile Strength (MPa)
PSO	0.0498	0.1721	1590	2.435	547.10
Experimental	0.05	0.17	1600	2.5	538
Firefly	0.0479	0.188	1718	1.36	904.37
Experimental	0.05	0.19	1700	1.4	910

## Appendix A: Pseudocode for Result Using PSO Algorithm

Start

NG = 1000    // Number of Generations

N = 100   // Number of Particles

D = 4         // Number of Input parameters

R []         // Input parameter values

RMax [], RMin []  //Maximum and Minimum value of the input parameter

VMax, VMin     //Velocity Maximum and Minimum value

PBest [], GBest    //Particle’s best value so far, Global best value.

f          // Objective function

P        // Represents a particle in N.

Step 1: Initialize the particles

 For I = 1 to d

 R [P] [i] = rand (RMin[i], RMax[i])

 V [P] [i] = rand (VMin[i], VMax[i])

Step 2: Apply Optimization Function given in Equation (3) and calculate fitness value.

 M [P] = f(R [P])

Step 3: if (M [P] >PBest [P]

 PBest [P] =M [P]

 If (M [P] >GBest)

 GBest = M [P]

Step 4: Repeat for i = 1 to NG

 Step 4.a:

  Find new velocity (V’) of each particle using Equation (1).

  Find the new R’ [P] [i] of each particle using Equation (2).

 Step 4.b:

  Calculate M’ [P] with R’ [P] [i] using Equation (3).

 Step 4.b:

  Replace PBest and GBest with M [P], when M [P] is maximum.

Step 5: GBest gives the optimal value.

End

## Appendix B: Pseudocode for Result Using Firefly algorithm

Start

N = 100   //Number of Generations

N = 20    //Number of fireflies

f       // Objective function in Eq.7

lower[],Upper[] //Array with Minimum bound value, maximum bound value of input parameters, respectively.

PR[][]   //Input parameter(CNT,Si3N3,depth,speed) values.

for k = 1 to n:

 Randomly populate PR[[] with values within lower[] and upper[]

 Calculate f[k] using Equation (7) with PR[] values.

Set BestFR = f [1]

While (numofgeneration<N)

for i = 1: n

for j = 1: n

Find f[i] and f[j] using Equation (7)

if (f (i) >f (j))

Compute distance d of the j-th butterfly from the i-th butterfly using Equation (3)

Compute the attractiveness value with the Equation (4)

Calculate the new parameter values applying the Equation (5)

If (new parameter values within the range)

Evaluate new solutions and update BestFR if needed

Else

Perform normalization using Equation (8)

 Evaluate new solutions and update BestFR if needed

 End if

Move the Best firefly randomly using Equation (6).

Evaluate new Best and update BestFR if needed.

End for j

End for i

End while

End
